# A one health perspective on the intestinal microbiome's role in COVID-19 outcomes and recovery

**DOI:** 10.3389/fcimb.2026.1763844

**Published:** 2026-05-19

**Authors:** Pallavi Singh, Anitha Saravanan, Jeanine Seitz, Nour Alkarzon, Nubwa Medugu

**Affiliations:** 1 Department of Biological Sciences, Northern Illinois University, DeKalb, IL, United States; 2 School of Nursing, Northern Illinois University, DeKalb, IL, United States; 3 Department of Medical Microbiology and Immunology, Nile University of Nigeria, Abuja, Nigeria

**Keywords:** gut dysbiosis, intestinal microbiome, microbiota-gut-brain axis (MGBA), One Health framework, SARS-CoV-2/COVID-19

## Abstract

Emerging infectious diseases, particularly zoonotic ones, remain major global health concerns. The Coronavirus Disease 2019 (COVID-19) pandemic, caused by Severe Acute Respiratory Syndrome Coronavirus-2 (SARS-CoV-2), highlights the interconnectedness of human, animal, and environmental health within the One Health framework. The intestinal microbiome plays a central role in host immunity and systemic homeostasis, and its disruption has been linked to altered disease severity and recovery patterns in COVID-19. Evidence suggests that SARS-CoV-2 infection induces intestinal dysbiosis, modifies immune signaling, and affects the microbiota-gut-brain axis (MGBA), contributing to neuropsychiatric and metabolic complications. This review synthesizes current findings on the intestinal microbiome’s role in COVID-19 pathophysiology and recovery, explores emerging therapeutic strategies including probiotics, prebiotics, and fecal microbiota transplantation, and emphasizes the importance of integrating microbiome research into pandemic preparedness through a One Health approach.

## 1 Introduction

A substantial body of research points to the role of zoonotic pathogens as major contributors to emerging and re-emerging infectious diseases (Kissler et al., 2020). The COVID-19 disease is caused by one such zoonotic pathogen, the novel SARS (Severe Acute Respiratory Syndrome)- CoV2 (coronavirus), which has been transmitted from its primary bat reservoir to humans (Shereen et al., 2020). Genetic analyses indicate that SARS-CoV-2 likely originated with possible spillover via an intermediate host such as the pangolin (Mahdy et al., 2020; Mallapaty, 2020). Therefore, the reemergence of SARS-CoV-2 was referred to as a ticking time bomb due to its large reservoir in bats by these studies (Cheng et al., 2007). The coronavirus research was initiated in the 1930s, which provided information such as on viral genome, infection cycle and immune response and offered leverage during the global pandemic to identify preventative and therapeutic strategies (Weiss, 2020). The rapid global spread of SARS-CoV-2 also spurred a new wave of investigations into viral transmission, pathogenesis, and host interactions. In addition to the immense data pool, the advent of time and cost-effective, high-throughput sequencing technologies provided genomic information at an unparalleled speed (Lu et al., 2020).

Additionally, numerous research groups tirelessly worked towards identifying SARS-CoV-2 mechanisms of action that lead to the havoc it causes on the host body. The severe clinical manifestations disproportionately affect different groups of people, such as the elderly (Wang et al., 2020). An increased presence of ACE-2 (Angiotensin converting enzyme) receptors and variation in HLA (Human Leukocyte Antigen) type were exhibited in patients (Muus et al., 2020; Nguyen et al., 2020). While initial attention focused on the respiratory system, it became increasingly clear that SARS-CoV-2 affects multiple organ systems, including the gastrointestinal (GI) tract, which expresses high levels of ACE2 (Lu et al., 2020; Muus et al., 2020). GI symptoms, fecal viral shedding, and prolonged alterations in bowel habits in some patients suggest a strong gut tropism. This raises critical questions about how SARS-CoV-2 may impact and be impacted by the host gut microbiome. However, the role of the enteric microbiome remains underappreciated.

The role of the intestinal microbial community finds its importance in enhanced immune support, curbing severe infections, and potential therapeutics, as well as in maintaining human host well-being. This narrative review synthesizes current evidence on SARS-CoV-2 and gut microbiota interactions, explores potential mechanisms linking dysbiosis to systemic and neuropsychiatric manifestations of COVID-19, and discusses microbiome-based therapeutic interventions. Here, we not only summarize recent advancements in SARS-CoV-2 as it relates to One-Health research but also the importance of host microbial communities as it relates to diagnosis, prevention, and therapeutics. We also outline future research priorities and call for a systems-level approach to pandemic preparedness that integrates microbiome science into host-pathogen-environment models.

Given the interdisciplinary nature of the topic, we included studies across microbiology, immunology, virology, gastroenterology, psychiatry, and public health. Relevant articles were identified through non-systematic literature searches in PubMed, Google Scholar, and Scopus, using combinations of keywords including COVID-19, SARS-CoV-2, gut microbiome, intestinal dysbiosis, ACE2, immune response, IL-18, long COVID, gut–brain axis, mental health, probiotics, prebiotics, fecal microbiota transplantation, and One Health. Literature published between January 2020 and October 2025 was prioritized, but foundational studies in microbiome-host interactions and coronavirus biology from earlier years were included where relevant. We included peer-reviewed original research articles, meta-analyses, narrative and systematic reviews, and key preprints that have had a substantial impact on the scientific understanding of COVID-19 and the microbiome. Reference lists of major review papers were also scanned for additional sources. Studies were selected for inclusion based on their relevance to the conceptual framework of the review, methodological rigor, and contribution to emerging understanding in the field. This narrative review synthesizes current evidence on SARS-CoV-2 and the gut microbiome, explores mechanisms linking microbial imbalance to immune dysregulation and neurobehavioral outcomes, and discusses microbiome-based therapeutic interventions. It also considers the relevance of these findings within a One Health framework, emphasizing the interdependence of human, animal, and environmental health.

## 2 SARS-CoV-2 characteristics and GI tropism

The Coronaviruses are a group of viruses which were initially thought to have relatively low pathogenicity until the 2002 SARS outbreak, when it caused the first 21st-century pandemic (Zhong et al., 2003). The family *Coronaviridae* consists of one of the largest groups of RNA viruses, characterized by being enveloped with blunt spikes protruding from the envelope.

### 2.1 Viral structure and entry mechanism

SARS-CoV-2 belongs to this *Coronaviridae* family, a group of enveloped, positive-sense, single-stranded RNA viruses characterized by their large genome (~30 kb) and distinctive spike (S) glycoproteins protruding from the viral envelope (Zhong et al., 2003; Fehr and Perlman, 2015). The genome of *Coronaviridae* is positive-sense and non-segmented with a 3’ poly A tail. Some genetic mutations involving coronaviruses of bats belong to the *Rhinolophus* genus (Ji et al., 2020; Mallapaty, 2020; Sharun et al., 2021). The genome consists of genes coding for structural and non-structural proteins. The replicase gene, which encodes non-structural proteins, occupies over half of the entire genome (about 20kb) (Zhong et al., 2003; Neuman et al., 2006). The genes encoding structural proteins are all preceded by sequences that mediate gene expression (transcriptional regulatory region) (Neuman et al., 2006). The Spike, Envelope, Membrane (M), and Nucleocapsid (N) regions follow sequentially in the genome. An untranslated region (UTR) is present at the 5’ end of the genome. The spikes are trimeric in form and protrude up to 20nm above the envelope, with each virion having an average of 75 spikes (Lai and Cavanagh, 1997; Neuman et al., 2006). The spike protein has a receptor-binding domain (S1), which mediates binding to host cell ACE2 (Mariano et al., 2020). During initiation of infection, the S1-ACE2 binding triggers a conformational change in S2, which precedes fusion of host-viral membranes. The envelop protein E is a small integral membrane protein composed of a short, hydrophilic amino end and a large hydrophobic transmembrane domain (Schoeman and Fielding, 2019). The E has been shown to influence virion morphogenesis and interactions with host cell signaling pathways (Boopathi et al., 2020). These structural features contribute to the virus’s infectivity and stability across different environments and tissue types.

### 2.2 Environmental stability and transmission potential

Time, surface type, pH - In autopsy models of human skin and mucus membranes, it has been reported that inactivation of SARS-CoV-2 takes 9 hours and 11 hours, respectively to get inactivated respectively with ethanol exposure of 15 seconds being sufficient to inactivate the virus on these surfaces (Hirose et al., 2021). In a study conducted at 50% relative humidity, it was found that on non-porous surfaces such as glass and polymer bank notes, the SARS-CoV-2 virus could be recovered five to seven days later at 20 °C, while on porous material such as paper notes at the same temperature, it persists for an average of nine days (Riddell et al., 2020). At higher environmental temperatures (40 °C) and 50% relative humidity, virus persistence reduces to five hours for paper notes, but is not significantly reduced for other tested non-porous surfaces (Riddell et al., 2020). The greatest temperature difference was seen for stainless steel, where virus persistence at 20 °C and 40 °C was greater than 28 days and less than two days, respectively (Riddell et al., 2020). The longer time of persistence on non-porous surfaces was similarly documented in other studies (Chin et al., 2020; Riddell et al., 2020).

### 2.3 ACE2 expression in the GI tract

While ACE2 is well known for its expression in lung alveolar epithelial cells, it is also highly expressed in the enterocytes of the small intestine (Lu et al., 2020). This widespread distribution suggests a dual role for ACE2 in both respiratory and GI infection pathways. ACE2 serves as the primary receptor for SARS-CoV-2 entry, mediating viral attachment through the S1 domain of the spike protein and facilitating membrane fusion via S2 (Lu et al., 2020). Both components have direct and indirect interactions with the gut microbiome, forming mechanistic links between viral entry, immune response, and microbial ecology. These expression patterns make the gut a plausible site of viral entry and replication. Indeed, gastrointestinal symptoms such as diarrhea, nausea, and abdominal discomfort are reported in a significant subset of COVID-19 patients, even in the absence of respiratory symptoms. Moreover, SARS-CoV-2 RNA has been detected in fecal samples of infected individuals, sometimes persisting for weeks after respiratory symptoms resolve (Zuo et al., 2020). The high expression of ACE2 in enterocytes suggests that the GI tract is not merely a secondary site of viral dissemination but may play an active role in viral replication and host immune response. Importantly, ACE2 is not only a receptor but also a regulator of amino acid transport and gut microbial homeostasis. It modulates the expression of the neutral amino acid transporter B^0AT1, which affects tryptophan absorption and downstream antimicrobial peptide production, factors essential for maintaining intestinal barrier integrity and microbial balance. This has important implications for both disease transmission and host–microbiota interactions, as viral infection may disrupt the intestinal barrier, alter microbial communities, and trigger local and systemic inflammation. Downregulation of ACE2 due to viral binding can thus impair gut homeostasis, increase permeability (“leaky gut”), and lead to dysbiosis, creating a feedback loop that enhances systemic inflammation and immune dysregulation.

## 3 The impact of COVID-19 on the intestinal microbiome

The GI microbial community consists of bacteria, fungi, eukaryotic microorganisms, and viruses. This microbial ecosystem plays essential roles in nutrient metabolism, mucosal barrier integrity, immune modulation, and protection against pathogens. In the context of viral infections, especially respiratory viruses, the gut microbiome can influence host susceptibility, immune responses, and clinical outcomes through bidirectional signaling across the gut–lung (GLAx) and microbiota-gut-brain axis (MGBA). SARS-CoV-2, with its documented tropism for intestinal tissues, has provided a new impetus to investigate how viral infection perturbs the gut microbiota and how these changes may affect disease trajectory. In this section, we summarize recent studies that have explored the bacterial community (bacteriome), the viral community (virome) of the gastrointestinal (GI) and the lungs. Further, we also emphasize the importance of the metabolites produced by these communities and their link to immunity in tackling COVID-19 disease.

### 3.1 Bacteriome-dysbiosis and disease severity

Recent studies have documented significant alterations in the composition of the gut bacterial community among patients with COVID-19. Through deep shotgun metagenomics study conducted by Zuo et al. on 15 patients with COVID-19 in Hong Kong found enhanced perturbation of the intestinal microbiome linked to increased severity of infections and SARS-CoV-2 fecal shedding (Zuo et al., 2020). Patients with no GI symptoms were found to have increased abundance of opportunistic pathogens such as *Clostridium hathewayi, Actinomyces viscosus*, and *Bacteroides nordii*, whereas a decrease in beneficial bacteria including *Faecalibacterium prausnitzii, Lachnospiraceae bacterium*, and *Eubacterium rectale* (Zuo et al., 2020). These alterations were associated with greater disease severity and prolonged fecal shedding of SARS-CoV-2 RNA. Similarly, a larger cohort study from China, inclusive of 62 COVID-19 patients, 33 seasonal flu patients, and 40 healthy controls, found an altered intestinal bacteriome by 16S rRNA gene analysis (Tao et al., 2020). This study, like the prior study, also reported an increased abundance of *Streptococcus, Clostridium, Lactobacillus*, and *Bifidobacterium*, whereas a decrease in the genera *Bacteroidetes, Roseburia, Faecalibacterium, Coprococcus*, in patients (Tao et al., 2020). Both studies suggested that the COVID-19 disease has long-term implications on the intestinal bacterial community and that this alteration is linked to an increase in severity of SARS-CoV-2 infection. These bacterial shifts may impair the host’s anti-inflammatory capacity and reduce short-chain fatty acid (SCFA) production, thereby weakening mucosal immunity and exacerbating systemic inflammation. In addition to these recent studies, several other studies have identified the importance of the gut microbiome in respiratory viral infection. They also suggest that manipulating the microbiome may be a possible strategy to manage COVID-19 disease.

### 3.2 The virome and GLAx

Although less extensively studied than the bacteriome, the human virome, including enteric and respiratory viruses, is increasingly recognized as a contributor to immune homeostasis and disease pathogenesis. SARS-CoV-2 infection may disturb the equilibrium of commensal viruses in both the gut and lungs, potentially altering host immune priming and antiviral responses. The extent to which virome shifts contribute to COVID-19 outcomes or influence the persistence of symptoms in long COVID remains an open question and a critical area for future research (Zuo et al., 2021; Xiang and Liu, 2022).

### 3.3 Systemic metabolites and immunity

The gut microbiome exerts far-reaching effects on systemic immunity through the production of microbial metabolites such as SCFAs, bile acids, and tryptophan catabolites. Disruption of the microbiome in COVID-19 patients has been associated with elevated levels of pro-inflammatory markers, including serum and fecal interleukin-18 (IL-18), suggesting a link between dysbiosis and cytokine-mediated tissue damage (Tao et al., 2020). Loss of SCFA-producing taxa may compromise epithelial barrier integrity and promote microbial translocation, further amplifying systemic inflammation. However, identification of differential microbial markers can support the host immune system development. These findings underscore the potential for microbiome-derived metabolites to serve as biomarkers of disease severity or targets for therapeutic intervention.

### 3.4 Microbiome–immune crosstalk in COVID-19

The severity and clinical course of COVID-19 vary widely among individuals, influenced by both viral characteristics and host factors. Among the most critical host elements implicated in SARS-CoV-2 susceptibility and disease progression are the expression of ACE2 and the genetic variability of HLA molecules. The HLA system plays a central role in antigen presentation and immune recognition of viral pathogens. Variation in HLA alleles across populations may explain differences in individual immune responses to SARS-CoV-2. Nguyen et al. identified specific HLA types associated with either increased susceptibility or potential protection against severe COVID-19 (Nguyen et al., 2020). Some alleles may bind and present SARS-CoV-2-derived peptides more efficiently, promoting stronger cytotoxic T-cell responses, while others may be less effective, allowing viral persistence and hyperinflammation. The gut microbiome may influence HLA-mediated responses by shaping the development and education of the immune system early in life and modulating T-cell differentiation during infection. Conversely, altered HLA expression during infection and inflammation may impact immune tolerance to commensal microbes, potentially worsening dysbiosis.

The interplay between ACE2, HLA variation, and the gut microbiota contributes to the heterogeneity of immune responses observed in COVID-19. Microbial metabolites such as short-chain fatty acids (SCFAs), bile acids, and indole derivatives influence innate and adaptive immunity, including regulatory T-cell (Treg) induction and cytokine production. Loss of key SCFA-producing bacteria during SARS-CoV-2 infection may reduce anti-inflammatory signaling, while expansion of pro-inflammatory microbes can amplify cytokine storms and tissue damage. Furthermore, elevated levels of IL-18, a cytokine linked to epithelial damage and inflammasome activation, have been observed in the serum and fecal samples of COVID-19 patients, suggesting microbial perturbations may directly affect systemic immune tone (Tao et al., 2020). Understanding these interconnected mechanisms is critical for identifying patients at higher risk for severe diseases and for developing microbiome-informed therapeutic strategies that support immune regulation and recovery.

## 4 Pandemic-led depression and interaction with the intestinal microbiome

The intestinal microbiome of a host plays an important role in the growth and development of the host. Recent studies have increasingly shown the importance of the gut microbiome in modulating the host behavior, including close social relationships, and have focused efforts on elucidating the gut-brain axis interaction. Social distancing and stress during the pandemic may impact microbiome diversity and composition, influencing mental health via the MGBA. Forecasts of deaths due to COVID-19 are predicted by several models built on levels of physical distancing (CDC, 2020). Social behaviors may influence MGBA, and studies that enhance our understanding of specific bacterial community members are important for understanding stress and anxiety as a result of physical distancing measures due to the current COVID-19 situation.

### 4.1 Microbiome and mental health impacts

In a functional sense, post SARS-CoV-2 infection, the cognitive state of an infected individual is significantly affected even after several months. Results from a six-month large-scale study published in 2021 demonstrate the impact of long COVID as compared to both flu-infected and uninfected cohorts (Al-Aly et al., 2021). Additional evidence from the National Alliance on Mental Illness (NAMI) and the Centers for Disease Control and Prevention (CDC) indicates rising depression and anxiety among adults and youth during the pandemic, potentially modulated by neuroimmune-microbiome pathways (CDC, 2020). COVID-positive individuals were shown to have increased mental health differences compared to the other two groups. Given that organ systems are interdependent and that dysregulation in one lead to dysregulation in others, the connection between altered states and longer-term outcomes post SARS-CoV-2 is unsurprising. The COVID-19 pandemic exerted profound psychological stress on global populations due to social isolation, economic instability, and uncertainty surrounding infection outcomes. Concurrently, evidence suggested that SARS-CoV-2 infection is associated with a wide range of neuropsychiatric complications, including anxiety, depression, cognitive dysfunction, and sleep disturbances, often persisting for months after recovery. These effects have been linked not only to systemic inflammation and neuro-invasion but also to disturbances in the MGBA mediated by the GI microbiome. Therefore, the increased rates of depression and anxiety during the COVID-19 pandemic, especially in vulnerable populations, stress the need for an improved mental healthcare system and access (Wickens et al., 2023).

### 4.2 The MGBA

The gut–brain axis represents a complex bidirectional network linking the central nervous system (CNS) and the enteric nervous system, with the gut microbiota playing a pivotal role in modulating this communication (Dinan and Cryan, 2017; Cryan et al., 2019). This communication occurs through neural, endocrine, immune, and metabolic pathways that collectively influence mood, cognition, and stress regulation. Commensal bacteria contribute to neurodevelopment and behavior through several mechanisms, including microbial synthesis of neurotransmitters such as γ-aminobutyric acid (GABA) and serotonin, modulation of the hypothalamic–pituitary–adrenal (HPA) axis, and immune–neuroendocrine signaling (Foster and McVey Neufeld, 2013; Cryan et al., 2019).

SARS-CoV-2 infection can disrupt this balance through intestinal dysbiosis, systemic inflammation, and direct effects on neural and vascular endothelium. Microbial dysbiosis resulting from SARS-CoV-2 infection may disrupt this axis, impair immune regulation, and contribute to neuroinflammation (Proal and VanElzakker, 2021). Reduced levels of *Faecalibacterium prausnitzii* and other short-chain fatty acid (SCFA)–producing taxa can diminish anti-inflammatory signaling, while the overgrowth of pathobionts may promote a pro-inflammatory milieu conducive to mood disturbances and cognitive decline (Miquel et al., 2013; Silva et al., 2020; Yeoh et al., 2021). These effects may be further amplified by social and environmental stressors experienced during the pandemic, which are known to influence the gut microbiome and stress-related neuroimmune pathways (Foster and McVey Neufeld, 2013; Hoban et al., 2018) The overgrowth of pathobionts further amplifies the production of lipopolysaccharides (LPS) and other pro-inflammatory metabolites that can cross the compromised gut–blood barrier and activate microglia within the CNS (Yang et al., 2015). Neuroimmune alterations in COVID-19 patients have been associated with fatigue, anxiety, cognitive impairment, and depressive symptoms persisting beyond viral clearance. These manifestations likely arise from a combination of cytokine dysregulation, altered tryptophan metabolism, and microbial metabolite imbalance. Reduced production of microbial-derived indoles, which normally act on the aryl hydrocarbon receptor (AhR), may weaken anti-inflammatory signaling in both the gut and brain. Social isolation, chronic stress, and pandemic-related lifestyle changes may further compound these biological effects, worsening the disruption of the MGBA. Overall, disturbances of the MGBA in COVID-19 reflect a convergence of microbial, immune, and psychosocial stressors. Understanding these interactions could inform adjunctive interventions, such as probiotics, prebiotics, or dietary modulation, designed to restore microbial and neuroimmune balance in affected individuals (Al-Aly et al., 2021).

### 4.3 Stress and MGBA

Physical distancing measures, while critical for infection control, have contributed to widespread psychological distress and behavioral changes, including altered diet, reduced physical activity, and disrupted sleep, factors known to influence gut microbiota composition. Animal models have shown that social isolation can reduce microbial diversity and increase the abundance of stress-associated taxa, providing a plausible biological link between pandemic-related behavioral changes and microbiome disruption. Forecasting models and epidemiological data from the CDC have revealed increased rates of anxiety and depressive disorders during the pandemic, with reported emergency department visits for mental health concerns rising by 24–31% in children and adolescents. In a large-scale retrospective study, three cohorts: individuals with SARS-CoV-2 infection, those infected with non-SARS-CoV-2 respiratory viruses (e.g., influenza), and uninfected controls exposed to the same environmental stressors (Al-Aly et al., 2021). Their findings demonstrated that individuals recovering from COVID-19 had significantly higher rates of mental health diagnoses, suggesting that SARS-CoV-2 infection may exert independent neuropsychiatric effects beyond contextual stressors. Additionally, case studies have reported notable correlations between COVID-associated mucormycosis, orbital exenteration, and psychological outcomes, including depression and reduced self-esteem (Ahuja et al., 2021). These findings, although preliminary, highlight the importance of understanding how infection-driven and behaviorally mediated microbiome alterations contribute to mental health.

### 4.4 Implications for long COVID and neuroimmune outcomes

Given the role of the microbiome in modulating systemic inflammation and neurocognitive health, persistent dysbiosis may contribute to long COVID symptoms such as “brain fog,” fatigue, and mood disorders (Proal and VanElzakker, 2021). While the precise mechanistic pathways remain to be elucidated, the convergence of microbial, immune, and neuroendocrine signaling pathways underscores the need to investigate microbiome-based interventions as part of comprehensive post-COVID recovery strategies (Silva et al., 2020; Proal and VanElzakker, 2021). Furthermore, the role of MGBA in neurodevelopment has also been reported, where disruptions in the GI microbiome may have critical consequences. GI microbiome differences have been noted in individuals with autism and schizophrenia (Wilson et al., 2024). Likewise, neuroinflammation associated with COVID-19, mediated by hypoxia and heightened cytokine production, may lead to encephalopathy (Duve et al., 2024). SARS-CoV2 induced excessive inflammatory response, activation of microglia and astrocytes, can damage the CNS and result in neurological symptoms. It is also hypothesized that cognitive decline and Alzheimer’s disease may be increased in individuals recovered from COVID-19 (Manosso et al., 2021). Collectively, this data supports the effects of SARS-CoV-2 on the nervous system with respect to mental health. Given what is already known about the anatomical and physiological link between the GI microbiome and the brain, continued investigation is warranted to study MGBA interaction in COVID-19.

## 5 One Health approach and host dynamism

The One Health approach emphasizes the interconnectedness of human, animal, and environmental health systems, recognizing that emerging infectious diseases are frequently driven by interactions at these interfaces. SARS-CoV-2 highlighted the importance of integrated surveillance across species boundaries and environmental reservoirs, demonstrating that pandemic preparedness cannot rely solely on human health systems (Destoumieux-Garzon et al., 2018; Mackenzie and Jeggo, 2019). Coordinated epidemiological efforts across medical, veterinary, and ecological disciplines are essential to detect spillover risks, monitor viral evolution, and implement strategic interventions. This approach provides a holistic framework to understand how microbial ecosystems and host dynamics shape disease risk and pathogen evolution (Leroy et al., 2020; Peccia et al., 2020). In practice, One Health strategies during COVID-19 included genomic surveillance of human and animal cases, wastewater-based epidemiology, and cross-sector collaboration on biosafety and zoonotic risk mitigation (Mackenzie and Jeggo, 2019; Jorwal et al., 2020; Ferri and Lloyd-Evans, 2021). These efforts demonstrated how environmental matrices, wildlife, and domestic animals can serve as sources or indicators of viral circulation. As such, advancing One Health infrastructure, particularly for genomic surveillance and cross-species monitoring, remains crucial for early warning systems and future pandemic prevention (Leroy et al., 2020).

### 5.1 Animal reservoir

Emerging evidence confirms that SARS-CoV-2 can infect a broad range of animal species, raising concern regarding wildlife reservoirs and the possibility of spillback into humans. Large-scale surveillance in North America identified widespread SARS-CoV-2 infection among white-tailed deer, with evidence of deer-to-deer transmission and unique evolutionary lineages persisting in animal hosts (Hale et al., 2022). Domestic pets and captive animals, including cats, tigers, and lions, have also acquired infection following human exposure (McAloose et al., 2020). These findings suggest viral adaptability across diverse mammalian hosts. Animal reservoirs pose risks for future re-emergence of viral variants, as demonstrated by mink farm outbreaks where SARS-CoV-2 transmission occurred from humans to mink and subsequently from mink back to humans (Oude Munnink et al., 2021). Wildlife and domestic reservoirs may act as “cryptic circulation” systems where lineage diversification occurs undetected. Therefore, sustained animal surveillance, enhanced biosecurity in domestic and farm environments, and integrated genomic systems are critical to mitigate zoonotic and reverse-zoonotic threats.

### 5.2 Human host and underlying conditions

Human susceptibility to severe COVID-19 is strongly influenced by pre-existing medical conditions, including diabetes, hypertension, cancer, and smoking-related disease. These conditions increase vulnerability through mechanisms such as chronic immune activation, endothelial dysfunction, impaired antiviral immunity, and metabolic dysregulation (Cuschieri and Grech, 2020; Zhou et al., 2020). Smoking further elevates risk by increasing ACE2 receptor expression in respiratory tissues, contributing to enhanced viral entry (Cai et al., 2020; Hussain et al., 2025). There is growing recognition that many of these comorbidities are linked to alterations in the gut microbiome, which regulates inflammation, metabolism, and immune responses. Metabolic diseases such as diabetes and hypertension are associated with gut dysbiosis and reduced short-chain-fatty-acid-producing microbes, which may predispose individuals to hyperinflammatory responses (He and Shi, 2017; Yang et al., 2021; Hou et al., 2022). Additionally, microbiome perturbations have been observed in patients with cancer undergoing immunotherapy, reflecting shared immune-microbiome pathways relevant to COVID-19 pathogenesis. As such, microbiome-targeted interventions may hold potential for improving host resilience and recovery in high-risk populations.

### 5.3 Transmission dynamics

SARS-CoV-2 transmission encompasses human-to-human spread, human-to-animal spillover, and animal-to-animal transmission, illustrating a dynamic ecological network. Although respiratory transmission is the predominant mechanism in humans, multiple reports document transmission from infected humans to domestic pets, mink, and zoo animals (McAloose et al., 2020). Wildlife infections, particularly in deer populations, suggest environmental and interspecies routes that can sustain viral spread outside human networks (Hale et al., 2022). Such cross-species infection pathways expand the viral evolutionary landscape and increase the risk of future emergence events (Oude Munnink et al., 2021). At the environmental interface, wastewater-based surveillance has proven to be an important tool for detecting viral RNA, monitoring infection trends, and identifying emerging variants (Peccia et al., 2020; Kirby et al., 2021). While surface transmission plays a minor role, wastewater monitoring highlights the environment as a valuable early-warning system for community transmission (Kirby et al., 2021). These findings underscore the role of microbes within interconnected ecological systems and the importance of integrated surveillance across human, animal, and environmental domains.

## 6 Clinical and therapeutic implications

Many laboratory-developed tests (LDTs) and commercial assays have emerged for diagnosing SARS-CoV-2 infection. Specimens such as nasal, nasopharyngeal, or oropharyngeal swabs, lower respiratory tract specimens such as sputum, tracheal aspirates, bronchoalveolar lavage, and nasopharyngeal wash can be tested. In addition to available vaccines for prevention, convalescent serum has been used to treat COVID-19. This passive antibody therapy involves the administration of antibodies against SARS-CoV-2; in contrast, active vaccination requires the induction of an immune response that takes time to develop and varies depending on the vaccine recipient. Likewise, repurposed drugs Remdesivir, Lopinavir/ritonavir, Chloroquin/hydrozycholoquin have also been implemented for COVID-19 treatment. However, these drugs can cause havoc on the body and the gut microbiome. Therefore, therapeutic interventions that enable the identification of beneficial gut microbes for a healthy immune system are of paramount importance. Furthermore, the gut microbiome has been reported to interact with the host cells through the production of metabolites that influence mitochondrial functions, which in turn can alter the immune response during a viral infection (Varshney et al., 2023).

### 6.1 Probiotics and prebiotics

Probiotics and prebiotics are two of the more accessible and practical ways to support the gut microbiome, especially during illness or recovery. Probiotics are the beneficial bacteria themselves, while prebiotics are the fibers and nutrients that help them grow and thrive. In the case of COVID-19, some studies have pointed to strains like *Lactobacillus rhamnosus* GG and *Bifidobacterium longum* as potentially helpful in reducing inflammation and easing respiratory symptoms (Baud et al., 2020; d'Ettorre et al., 2020). Prebiotics such as inulin feed good bacteria and help promote the production of short-chain fatty acids (SCFAs), which are linked to immune balance and gut barrier health (Barber et al., 2020). While the research is still early, especially when it comes to COVID-specific outcomes, there’s some promising evidence that these supplements could be useful for people dealing with lingering GI symptoms or long COVID. That said, responses can vary a lot depending on the individual and the specific strains or types used, so there’s definitely a need for more clinical trials. Future pandemics and Prevention.

### 6.2 Fecal microbiota transplantation

Fecal microbiota transplantation (FMT), while invasive, is an established therapy for recurrent *Clostridioides difficile* infection and is being investigated for microbiome restoration in post-COVID dysbiosis. In FNT, stools are transplanted from a healthy donor into the gut of someone whose microbiome has been disrupted. The goal is to bring back microbial diversity and reduce inflammation. A small pilot study even found some improvement in post-COVID symptoms and microbiome diversity after FMT (Hazan et al., 2022). It’s a fascinating area, but still very experimental when it comes to COVID. There are safety concerns, too, especially around screening donors for viruses like SARS-CoV-2. So while FMT has potential, it’s not ready for mainstream use in this setting without larger, more rigorous studies (Ianiro et al., 2014).

### 6.3 Dietary interventions and lifestyle

It is well-established that diet and lifestyle influence the gut microbiome, with diets rich in fiber, fruits, vegetables, fermented foods, and healthy fats (mirroring patterns such as the Mediterranean or DASH diets) promoting a more diverse and balanced microbiome, which supports immune homeostasis (Merino et al., 2021; Wastyk et al., 2021; Venter et al., 2022; Perler et al., 2023). This is important because the GI microbiome plays a role in regulating the body’s immune response, which can influence recovery from infections. For example, a human trial found that both high-fiber and fermented-food interventions altered gut microbial composition and reduced inflammatory immune markers (Wastyk et al., 2021). Moreover, pandemic-related lifestyle changes, including increased stress, poor sleep, reduced physical activity, and higher intake of processed foods, may have further disrupted gut microbial balance. Such lifestyle changes can further change the microbiome causing dysbiosis (Robertson et al., 2019). Encouraging healthier habits during recovery isn’t just good general advice; emerging evidence suggests that optimizing the microbiome could help reduce lingering symptoms and speed recovery (Venter et al., 2022; Perler et al., 2023).

### 6.4 Microbiome-based biomarkers and personalized therapy

One of the more exciting areas of research is the potential for individual gut microbiome signatures to serve as biomarkers and guide therapy. Several studies have observed that patients with COVID-19 who have lower levels of short-chain-fatty-acid (SCFA)-producing bacteria (such as *Faecalibacterium prausnitzii* or *Eubacterium rectale*) or disrupted microbial diversity tend to have worse disease outcomes or more persistent (“long COVID”) symptoms (Tao et al., 2020; Yeoh et al., 2021; Zhang et al., 2022). Due to advances in sequencing, metabolomics, and data science, researchers are increasingly able to identify microbial “signatures” and host-microbe interactions. It’s still early days, but using gut data to guide treatment could be a game-changer for how we approach recovery and long-term health after COVID-19. In the future, these insights may open the door to personalized interventions (Crisci et al., 2020). For example, tailored probiotic blends or diet plans based on an individual’s microbiome profile can offer a more precision-based approach to recovery and long-term health after COVID-19 (Lim et al., 2025).

## 7 Current limitations and conclusion

Research on the interaction between *SARS-CoV-2* infection and the intestinal microbiome has expanded rapidly, yet several methodological and conceptual limitations persist. First, most available studies are cross-sectional and involve small sample sizes, which restrict causal inference. The heterogeneity in study populations, analytical methods, and sequencing platforms further complicates data comparability across cohorts (Tao et al., 2020). Many investigations rely on 16S rRNA sequencing, which lacks the taxonomic and functional resolution necessary to identify strain-level differences or to capture changes in the virome and mycobiome. Additionally, limited attention has been given to confounding variables such as antibiotic exposure, diet, and disease severity, which all influence microbial diversity and composition.

Another limitation is the underrepresentation of longitudinal studies that follow patients from acute infection through recovery. Such designs are crucial for distinguishing transient dysbiosis from persistent microbiome alterations associated with long COVID. Similarly, the environmental and animal components of the One Health framework remain underexplored. Very few studies have examined how microbial and viral exchanges between humans, animals, and the environment shape COVID-19 dynamics or influence antimicrobial resistance. Integrating these perspectives will be key to developing a systems-level understanding of the pandemic’s ecological context (Budden et al., 2017).

Therapeutically, the evidence supporting microbiome-based interventions, such as probiotics, prebiotics, dietary modulation, and fecal microbiota transplantation, remains preliminary. Most clinical trials are either small-scale or open-label and lack standardized endpoints. As a result, while microbiome modulation holds promise as an adjunctive strategy for COVID-19 management and recovery, more rigorous randomized controlled trials are needed to confirm efficacy and safety.

In conclusion, the interplay between SARS-CoV-2, the gut microbiome, and host immunity represents an evolving frontier in infectious disease research. Alterations in gut microbial communities can influence immune, metabolic, and neuropsychiatric outcomes, offering a biological basis for the diverse manifestations of COVID-19. A One Health approach, integrating human, animal, and environmental microbiome data, will be essential for pandemic preparedness and for anticipating future zoonotic threats. Advances in multi-omics technologies, longitudinal cohort studies, and integrative bioinformatics are likely to refine our understanding of microbial–host interactions and identify novel targets for prevention and therapy. Continued interdisciplinary research will determine whether restoring microbiome balance can mitigate both acute infection and long-term sequelae of COVID-19 (Cryan et al., 2019).

## References

[B1] Ahuja A. Samudra M. Prasad S. P. Chaudhury S. Bora S. Singh V. . (2021). Correlates of depression, anxiety, self-esteem, and suicidal ideas in COVID-associated mucormycosis patients and the effects of treatment. Ind. Psychiatry J. 30, S75–S82. doi: 10.4103/0972-6748.328863. PMID: 34908669 PMC8611535

[B2] Al-Aly Z. Xie Y. Bowe B. (2021). High-dimensional characterization of post-acute sequelae of COVID-19. Nature 594, 259–264. doi: 10.1038/s41586-021-03553-9. PMID: 33887749

[B3] Barber T. M. Kabisch S. Pfeiffer A. F. H. Weickert M. O. (2020). The health benefits of dietary fibre. Nutrients 12 (10), 3209. doi: 10.3390/nu12103209. PMID: 33096647 PMC7589116

[B4] Baud D. Dimopoulou Agri V. Gibson G. R. Reid G. Giannoni E. (2020). Using probiotics to flatten the curve of coronavirus disease COVID-2019 pandemic. Front. Public Health 8, 186. doi: 10.3389/fpubh.2020.00186. PMID: 32574290 PMC7227397

[B5] Boopathi S. Poma A. B. Kolandaivel P. (2020). Novel 2019 coronavirus structure, mechanism of action, antiviral drug promises and rule out against its treatment. J. Biomol. Struct. Dyn. 39 (9), 3409–3418. doi: 10.1080/07391102.2020.1758788. PMID: 32306836 PMC7196923

[B6] Budden K. F. Gellatly S. L. Wood D. L. Cooper M. A. Morrison M. Hugenholtz P. . (2017). Emerging pathogenic links between microbiota and the gut-lung axis. Nat. Rev. Microbiol. 15, 55–63. doi: 10.1038/nrmicro.2016.142. PMID: 27694885

[B7] Cai G. Bosse Y. Xiao F. Kheradmand F. Amos C. I. (2020). Tobacco smoking increases the lung gene expression of ACE2, the receptor of SARS-CoV-2. Am. J. Respir. Crit. Care Med. 201, 1557–1559. doi: 10.1164/rccm.202003-0693le. PMID: 32329629 PMC7301735

[B8] CDC (2020). COVID-19 forecasts: deaths 2020. Available online at: https://www.cdc.gov/coronavirus/2019-ncov/covid-data/forecasting-us.html (Accessed September, 2024).

[B9] Cheng V. C. Lau S. K. Woo P. C. Yuen K. Y. (2007). Severe acute respiratory syndrome coronavirus as an agent of emerging and reemerging infection. Clin. Microbiol. Rev. 20, 660–694. doi: 10.1128/cmr.00023-07. PMID: 17934078 PMC2176051

[B10] Chin A. W. H. Chu J. T. S. Perera M. R. A. Hui K. P. Y. Yen H.-L. Chan M. C. W. . (2020). Stability of SARS-CoV-2 in different environmental conditions. Lancet Microbe 1. doi: 10.1016/s2666-5247(20)30095-1. PMID: 32835322 PMC7214863

[B11] Crisci C. D. Ardusso L. R. F. Mossuz A. Muller L. (2020). A precision medicine approach to SARS-CoV-2 pandemic management. Curr. Treat. Options Allergy 7, 422–440. doi: 10.1007/s40521-020-00258-8. PMID: 32391242 PMC7205603

[B12] Cryan J. F. O'Riordan K. J. Cowan C. S. M. Sandhu K. V. Bastiaanssen T. F. S. Boehme M. . (2019). The microbiota-gut-brain axis. Physiol. Rev. 99, 1877–2013. doi: 10.1177/0706743716635538. PMID: 31460832

[B13] Cuschieri S. Grech S. (2020). COVID-19: a one-way ticket to a global childhood obesity crisis? J. Diabetes Metab. Disord. 19, 2027–2030. doi: 10.1007/s40200-020-00682-2. PMID: 33173756 PMC7644278

[B14] d'Ettorre G. Ceccarelli G. Marazzato M. Campagna G. Pinacchio C. Alessandri F. . (2020). Challenges in the management of SARS-CoV2 infection: the role of oral bacteriotherapy as complementary therapeutic strategy to avoid the progression of COVID-19. Front. Med. (Lausanne) 7, 389. doi: 10.3389/fmed.2020.00389 32733907 PMC7358304

[B15] Destoumieux-Garzon D. Mavingui P. Boetsch G. Boissier J. Darriet F. Duboz P. . (2018). The one health concept: 10 years old and a long road ahead. Front. Vet. Sci. 5, 14. doi: 10.3389/fvets.2018.00014. PMID: 29484301 PMC5816263

[B16] Dinan T. G. Cryan J. F. (2017). The microbiome-gut-brain axis in health and disease. Gastroenterol. Clin. North. Am. 46, 77–89. doi: 10.1016/j.gtc.2016.09.007. PMID: 28164854

[B17] Duve K. Petakh P. Kamyshnyi O. (2024). COVID-19-associated encephalopathy: connection between neuroinflammation and microbiota-gut-brain axis. Front. Microbiol. 15, 1406874. doi: 10.3389/fmicb.2024.1406874. PMID: 38863751 PMC11165208

[B18] Fehr A. R. Perlman S. (2015). “ Coronaviruses: An overview of their replication and pathogenesis,” in Coronaviruses. Methods in Molecular Biology. 1282, 1–23. doi: 10.1007/978-1-4939-2438-7_1 25720466 PMC4369385

[B19] Ferri M. Lloyd-Evans M. (2021). The contribution of veterinary public health to the management of the COVID-19 pandemic from a one health perspective. One Health 12, 100230. doi: 10.1016/j.onehlt.2021.100230. PMID: 33681446 PMC7912361

[B20] Foster J. A. McVey Neufeld K. A. (2013). Gut-brain axis: how the microbiome influences anxiety and depression. Trends Neurosci. 36, 305–312. doi: 10.1016/j.tins.2013.01.005. PMID: 23384445

[B21] Hale V. L. Dennis P. M. McBride D. S. Nolting J. M. Madden C. Huey D. . (2022). SARS-CoV-2 infection in free-ranging white-tailed deer. Nature 602, 481–486. doi: 10.1038/s41586-021-04353-x. PMID: 34942632 PMC8857059

[B22] Hazan S. Stollman N. Bozkurt H. S. Dave S. Papoutsis A. J. Daniels J. . (2022). Lost microbes of COVID-19: Bifidobacterium, Faecalibacterium depletion and decreased microbiome diversity associated with SARS-CoV-2 infection severity. BMJ Open Gastroenterol. 9, e000871. doi: 10.1136/bmjgast-2022-000871. PMID: 35483736 PMC9051551

[B23] He M. Shi B. (2017). Gut microbiota as a potential target of metabolic syndrome: the role of probiotics and prebiotics. Cell Biosci. 7, 54. doi: 10.1186/s13578-017-0183-1. PMID: 29090088 PMC5655955

[B24] Hirose R. Ikegaya H. Naito Y. Watanabe N. Yoshida T. Bandou R. . (2021). Survival of severe acute respiratory syndrome coronavirus 2 (SARS-CoV-2) and influenza virus on human skin: Importance of hand hygiene in coronavirus disease 2019 (COVID-19). Clin. Infect. Dis. 73, e4329–e4335. doi: 10.1093/cid/ciaa1517. PMID: 33009907 PMC7665347

[B25] Hoban A. E. Stilling R. M. Moloney G. Shanahan F. Dinan T. G. Clarke G. . (2018). The microbiome regulates amygdala-dependent fear recall. Mol. Psychiatry 23, 1134–1144. doi: 10.1038/mp.2017.100. PMID: 28507320 PMC5984090

[B26] Hou K. Wu Z. X. Chen X. Y. Wang J. Q. Zhang D. Xiao C. . (2022). Microbiota in health and diseases. Signal. Transduct Target Ther. 7, 135. doi: 10.1038/s41392-022-00974-4. PMID: 35461318 PMC9034083

[B27] Hussain S. S. Libby E. F. Peabody Lever J. E. Tipper J. L. Phillips S. E. Mazur M. . (2025). ACE-2 blockade and TMPRSS2 inhibition mitigate SARS-CoV-2 severity following cigarette smoke exposure in airway epithelial cells *in vitro*. Mol. Ther. Nucleic Acids 36, 102580. doi: 10.1016/j.omtn.2025.102580. PMID: 40612711 PMC12221736

[B28] Ianiro G. Bibbo S. Gasbarrini A. Cammarota G. (2014). Therapeutic modulation of gut microbiota: current clinical applications and future perspectives. Curr. Drug Targets 15, 762–770. doi: 10.2174/1389450115666140606111402. PMID: 24909808

[B29] Ji W. Wang W. Zhao X. Zai J. Li X. (2020). Cross-species transmission of the newly identified coronavirus 2019-nCoV. J. Med. Virol. 92, 433–440. doi: 10.1002/jmv.25682. PMID: 31967321 PMC7138088

[B30] Jorwal P. Bharadwaj S. Jorwal P. (2020). One health approach and COVID-19: A perspective. J. Family Med. Prim Care 9, 5888–5891. doi: 10.4103/jfmpc.jfmpc_1058_20. PMID: 33681013 PMC7928117

[B31] Kirby A. E. Walters M. S. Jennings W. C. Fugitt R. LaCross N. Mattioli M. . (2021). Using wastewater surveillance data to support the COVID-19 response - United States, 2020-2021. MMWR Morb Mortal Wkly Rep. 70, 1242–1244. doi: 10.15585/mmwr.mm7036a2. PMID: 34499630 PMC8437053

[B32] Kissler S. M. Tedijanto C. Goldstein E. Grad Y. H. Lipsitch M. (2020). Projecting the transmission dynamics of SARS-CoV-2 through the postpandemic period. Science. 368, 860–868. doi: 10.1126/science.abb5793. PMID: 32291278 PMC7164482

[B33] Lai M. M. C. Cavanagh D. (1997). “ The molecular biology of coronaviruses,” in Advances in Virus Research. 48, 1–100. doi: 10.1016/S0065-3527(08)60286-9 9233431 PMC7130985

[B34] Leroy E. M. Ar Gouilh M. Brugere-Picoux J. (2020). The risk of SARS-CoV-2 transmission to pets and other wild and domestic animals strongly mandates a one-health strategy to control the COVID-19 pandemic. One Health 10, 100133. doi: 10.1016/j.onehlt.2020.100133. PMID: 32363229 PMC7194722

[B35] Lim H. X. Khalid K. Abdullah A. D. I. Lee L. H. Raja Ali R. A. (2025). Subphenotypes of long COVID and the clinical applications of probiotics. Biomed. Pharmacother. 183, 117855. doi: 10.1016/j.biopha.2025.117855. PMID: 39862702

[B36] Lu R. Zhao X. Li J. Niu P. Yang B. Wu H. . (2020). Genomic characterisation and epidemiology of 2019 novel coronavirus: implications for virus origins and receptor binding. Lancet 395, 565–574. doi: 10.1016/s0140-6736(20)30251-8. PMID: 32007145 PMC7159086

[B37] Mackenzie J. S. Jeggo M. (2019). The one health approach-why is it so important? Trop. Med. Infect. Dis. 4(2), 88. doi: 10.3390/tropicalmed4020088. PMID: 31159338 PMC6630404

[B38] Mahdy M. A. A. Younis W. Ewaida Z. (2020). An overview of SARS-CoV-2 and animal infection. Front. Vet. Sci. 7, 596391. doi: 10.3389/fvets.2020.596391. PMID: 33363234 PMC7759518

[B39] Mallapaty S. (2020). Animal source of the coronavirus continues to elude scientists. Nature. doi: 10.1038/d41586-020-01449-8. PMID: 32427902

[B40] Manosso L. M. Arent C. O. Borba L. A. Ceretta L. B. Quevedo J. Reus G. Z. (2021). Microbiota-gut-brain communication in the SARS-CoV-2 infection. Cells 10(8), 1993. doi: 10.3390/cells10081993. PMID: 34440767 PMC8391332

[B41] Mariano G. Farthing R. J. Lale-Farjat S. L. M. Bergeron J. R. C. (2020). Structural characterization of SARS-CoV-2: Where we are, and where we need to be. Front. Mol. Biosci. 7. doi: 10.3389/fmolb.2020.605236. PMID: 33392262 PMC7773825

[B42] McAloose D. Laverack M. Wang L. Killian M. L. Caserta L. C. Yuan F. . (2020). From people to Panthera: Natural SARS-CoV-2 infection in tigers and lions at the Bronx Zoo. mBio 11. doi: 10.1128/mbio.02220-20. PMID: 33051368 PMC7554670

[B43] Merino J. Joshi A. D. Nguyen L. H. Leeming E. R. Mazidi M. Drew D. A. . (2021). Diet quality and risk and severity of COVID-19: a prospective cohort study. Gut 70, 2096–2104. doi: 10.1136/gutjnl-2021-325353. PMID: 34489306 PMC8500931

[B44] Miquel S. Martin R. Rossi O. Bermudez-Humaran L. G. Chatel J. M. Sokol H. . (2013). Faecalibacterium prausnitzii and human intestinal health. Curr. Opin. Microbiol. 16, 255–261. doi: 10.1016/j.mib.2013.06.003. PMID: 23831042

[B45] Muus C. Luecken M. D. Eraslan G. Waghray A. Heimberg G. Sikkema L. . (2020). Integrated analyses of single-cell atlases reveal age, gender, and smoking status associations with cell type-specific expression of mediators of SARS-CoV-2 viral entry and highlights inflammatory programs in putative target cells. bioRxiv. doi: 10.1101/2020.04.19.049254

[B46] Neuman B. W. Adair B. D. Yoshioka C. Quispe J. D. Orca G. Kuhn P. . (2006). Supramolecular architecture of severe acute respiratory syndrome coronavirus revealed by electron cryomicroscopy. J. Virol. 80, 7918–7928. doi: 10.1128/jvi.00645-06. PMID: 16873249 PMC1563832

[B47] Nguyen A. David J. K. Maden S. K. Wood M. A. Weeder B. R. Nellore A. . (2020). Human leukocyte antigen susceptibility map for SARS-CoV-2. J. Virol. 94, 10.1128/jvi.00510-20. doi: 10.1128/jvi.00510-20. PMID: 32303592 PMC7307149

[B48] Oude Munnink B. B. Sikkema R. S. Nieuwenhuijse D. F. Molenaar R. J. Munger E. Molenkamp R. . (2021). Transmission of SARS-CoV-2 on mink farms between humans and mink and back to humans. Science 371, 172–177. doi: 10.1126/science.abe5901. PMID: 33172935 PMC7857398

[B49] Peccia J. Zulli A. Brackney D. E. Grubaugh N. D. Kaplan E. H. Casanovas-Massana A. . (2020). Measurement of SARS-CoV-2 RNA in wastewater tracks community infection dynamics. Nat. Biotechnol. 38, 1164–1167. doi: 10.1038/s41587-020-0684-z. PMID: 32948856 PMC8325066

[B50] Perler B. K. Friedman E. S. Wu G. D. (2023). The role of the gut microbiota in the relationship between diet and human health. Annu. Rev. Physiol. 85, 449–468. doi: 10.1146/annurev-physiol-031522-092054. PMID: 36375468

[B51] Proal A. D. VanElzakker M. B. (2021). Long COVID or post-acute sequelae of COVID-19 (PASC): An overview of biological factors that may contribute to persistent symptoms. Front. Microbiol. 12, 698169. doi: 10.3389/fmicb.2021.698169. PMID: 34248921 PMC8260991

[B52] Riddell S. Goldie S. Hill A. Eagles D. Drew T. W. (2020). The effect of temperature on persistence of SARS-CoV-2 on common surfaces. Virol. J. 17, 145. doi: 10.1186/s12985-020-01418-7. PMID: 33028356 PMC7538848

[B53] Robertson R. C. Manges A. R. Finlay B. B. Prendergast A. J. (2019). The human microbiome and child growth - first 1000 days and beyond. Trends Microbiol. 27, 131–147. doi: 10.1016/j.tim.2018.09.008. PMID: 30529020

[B54] Schoeman D. Fielding B. C. (2019). Coronavirus envelope protein: current knowledge. Virol. J. 16, 69. doi: 10.1186/s12985-019-1182-0. PMID: 31133031 PMC6537279

[B55] Sharun K. Dhama K. Pawde A. M. Gortazar C. Tiwari R. Bonilla-Aldana D. K. . (2021). SARS-CoV-2 in animals: potential for unknown reservoir hosts and public health implications. Vet. Q 41, 181–201. doi: 10.1080/01652176.2021.1921311. PMID: 33892621 PMC8128218

[B56] Shereen M. A. Khan S. Kazmi A. Bashir N. Siddique R. (2020). COVID-19 infection: Origin, transmission, and characteristics of human coronaviruses. J. Adv. Res. 24, 91–98. doi: 10.1016/j.jare.2020.03.005. PMID: 32257431 PMC7113610

[B57] Silva Y. P. Bernardi A. Frozza R. L. (2020). The role of short-chain fatty acids from gut microbiota in gut-brain communication. Front. Endocrinol. (Lausanne) 11, 25. doi: 10.3389/fendo.2020.00025. PMID: 32082260 PMC7005631

[B58] Tao W. Zhang G. Wang X. Guo M. Zeng W. Xu Z. . (2020). Analysis of the intestinal microbiota in COVID-19 patients and its correlation with the inflammatory factor IL-18. Med. Microecol. 5, 100023. doi: 10.1016/j.medmic.2020.100023. PMID: 34173452 PMC7832617

[B59] Varshney V. Kushwah P. S. Agrawal N. Goyal A. Singh G. (2023). Cellular and molecular insights into microbiota-mitochondria interplay, therapeutic biomarkers and interventional approaches in COVID-19: a review. Biocell 47, 2141–2149. doi: 10.32604/biocell.2023.030853

[B60] Venter C. Meyer R. W. Greenhawt M. Pali-Scholl I. Nwaru B. Roduit C. . (2022). Role of dietary fiber in promoting immune health-An EAACI position paper. Allergy 77, 3185–3198. doi: 10.22541/au.163807080.07633496/v1. PMID: 35801383

[B61] Wang L. He W. Yu X. Hu D. Bao M. Liu H. . (2020). Coronavirus disease 2019 in elderly patients: Characteristics and prognostic factors based on 4-week follow-up. J. Infect. 80 (6), 639–645. doi: 10.1016/j.jinf.2020.03.019. PMID: 32240670 PMC7118526

[B62] Wastyk H. C. Fragiadakis G. K. Perelman D. Dahan D. Merrill B. D. Yu F. B. . (2021). Gut-microbiota-targeted diets modulate human immune status. Cell 184, 4137–4153. doi: 10.1016/j.cell.2021.06.019. PMID: 34256014 PMC9020749

[B63] Weiss S. R. (2020). Forty years with coronaviruses. J. Exp. Med. 217 (5), e20200537. doi: 10.1084/jem.20200537. PMID: 32232339 PMC7103766

[B64] Wickens C. M. Popal V. Fecteau V. Amoroso C. Stoduto G. Rodak T. . (2023). The mental health impacts of the COVID-19 pandemic among individuals with depressive, anxiety, and stressor-related disorders: A scoping review. PloS One 18, e0295496. doi: 10.1371/journal.pone.0295496. PMID: 38096173 PMC10721054

[B65] Wilson J. D. Dworsky-Fried M. Ismail N. (2024). Neurodevelopmental implications of COVID-19-induced gut microbiome dysbiosis in pregnant women. J. Reprod. Immunol. 165, 104300. doi: 10.1016/j.jri.2024.104300. PMID: 39004033

[B66] Xiang H. Liu Q. P. (2022). Alterations of the gut microbiota in coronavirus disease 2019 and its therapeutic potential. World J. Gastroenterol. 28, 6689–6701. doi: 10.3748/wjg.v28.i47.6689. PMID: 36620345 PMC9813939

[B67] Yang T. Santisteban M. M. Rodriguez V. Li E. Ahmari N. Carvajal J. M. . (2015). Gut dysbiosis is linked to hypertension. Hypertension 65, 1331–1340. doi: 10.1161/hypertensionaha.115.05315. PMID: 25870193 PMC4433416

[B68] Yang G. Wei J. Liu P. Zhang Q. Tian Y. Hou G. . (2021). Role of the gut microbiota in type 2 diabetes and related diseases. Metabolism 117, 154712. doi: 10.1016/j.metabol.2021.154712. PMID: 33497712

[B69] Yeoh Y. K. Zuo T. Lui G. C. Zhang F. Liu Q. Li A. Y. . (2021). Gut microbiota composition reflects disease severity and dysfunctional immune responses in patients with COVID-19. Gut 70, 698–706. doi: 10.1136/gutjnl-2020-323020. PMID: 33431578 PMC7804842

[B70] Zhang F. Wan Y. Zuo T. Yeoh Y. K. Liu Q. Zhang L. . (2022). Prolonged impairment of short-chain fatty acid and L-isoleucine biosynthesis in gut microbiome in patients with COVID-19. Gastroenterology 162, 548–561. doi: 10.1053/j.gastro.2021.10.013. PMID: 34687739 PMC8529231

[B71] Zhong N. S. Zheng B. J. Li Y. M. Poon L. L. M. Xie Z. H. Chan K. H. . (2003). Epidemiology and cause of severe acute respiratory syndrome (SARS) in Guangdong, People's Republic of China, in February, 2003. Lancet 362, 1353–1358. doi: 10.1016/s0140-6736(03)14630-2. PMID: 14585636 PMC7112415

[B72] Zhou F. Yu T. Du R. Fan G. Liu Y. Liu Z. . (2020). Clinical course and risk factors for mortality of adult inpatients with COVID-19 in Wuhan, China: a retrospective cohort study. Lancet 395, 1054–1062. doi: 10.1016/s0140-6736(20)30566-3. PMID: 32171076 PMC7270627

[B73] Zuo T. Liu Q. Zhang F. Yeoh Y. K. Wan Y. Zhan H. . (2021). Temporal landscape of human gut RNA and DNA virome in SARS-CoV-2 infection and severity. Microbiome 9, 91. doi: 10.1186/s40168-021-01008-x. PMID: 33853691 PMC8044506

[B74] Zuo T. Zhang F. Lui G. C. Y. Yeoh Y. K. Li A. Y. L. Zhan H. . (2020). Alterations in gut microbiota of patients with COVID-19 during time of hospitalization. Gastroenterology 159, 944–955. doi: 10.1053/j.gastro.2020.05.048. PMID: 32442562 PMC7237927

